# Competition between local disordering and global ordering fields in nematic liquid crystals

**DOI:** 10.3762/bjoc.6.2

**Published:** 2010-01-07

**Authors:** Matej Cvetko, Milan Ambrožič, Samo Kralj

**Affiliations:** 1 Regional Development Agency Mura Ltd, Lendavska 5a, 9000 Murska Sobota, Slovenia; 2 Laboratory of Physics of Complex Systems, Faculty of Natural Sciences and Mathematics, University of Maribor, Koroška 160, 2000 Maribor, Slovenia; 3 Condensed Matter Physics Department, Jožef Stefan Institute, Jamova 39, 1000 Ljubljana, Slovenia

**Keywords:** disorder, Imry-Ma theorem, liquid crystals, memory effect, orientational order

## Abstract

We study the influence of external electric or magnetic field *B* on orientational ordering of nematic liquid crystals or of other rod-like objects (e.g. nanotubes immersed in a liquid) in the presence of random anisotropy field type of disorder. The Lebwohl–Lasher lattice type of semi-microscopic approach is used at zero temperature. Therefore, results are valid well below the transition into the isotropic phase. We calculate the correlation function of systems as a function of *B*, concentration *p* of impurities imposing random anisotropy field disorder, the disorder strength *W* and system dimensionality (2D and 3D systems). In order to probe memory effects we calculate correlation length *ξ* for random and homogeneous initial configurations. We determine the crossover fields *B*_c_(*p*) separating roughly the ordered and disordered regime. Memory effects are apparent only in the latter case, i.e. for *B* < *B*_c_.

**PACS numbers**: 47.51.+a, 47.54.-r, 07.05.Tp, 61.30.-v

## Introduction

For years there has been a strong interest in the phase and structural behavior of randomly perturbed liquid crystals (LCs) [1]. Such systems could be used in various electro-optical applications. On the other hand they represent also an adequate testing ground [2] to study fundamental questions concerning the impact of disorder [3–6] on various phase and structural transitions.

Most studies so far have been carried out in thermotropic nematic LC phases, which exhibit long range orientational order [7]. To enforce disorder to LC ordering one either confines LC to various porous matrices [8–12] (e.g., aerogels, Controlled-pore glass, Vycor glass) or mixes LCs with nanoparticles. For the latter purpose aerosil nanoparticles [13–15] are particularly adequate. They form random networks, the structure of which can be altered by varying the concentration of nanoparticles. At least three qualitatively different regimes can be realized [16].

Studies so far have mainly focused on structural and phase behavior [8–18]. It has been shown that the isotropic nematic phase transition is typically replaced by the paranematic–nematic (PN–N) phase transition. The transition temperature in most cases decreases with increased disorder strength. If disorder is strong enough the transition can disappear. In the nematic phase memory effects can be observed revealing to some extent glass-like features.

To our knowledge none of the studies so far have systematically explored the effect of external ordering field (*B*) in such systems. This is the topic of our paper. We consider the competition between local disordering fields and the global external magnetic or electric ordering field. Local random fields can be in practice imposed geometrically. Experimental examples are LCs confined to a porous matrix [1], mixtures of LCs and aerosil nanoparticles [12–15], binary mixtures of different rodlike objects which tend to be oriented perpendicularly [19], and nanotubes immersed in liquid crystals [20–21]. We focus on *B* induced erasing of memory effects in such systems using the Lebwohl–Lasher [22] type lattice model deep in the nematic phase.

The structure of the article is as follows. First we present the semi-microscopic lattice model that we use. Then the results are presented and discussed. In the following section we summarize our results. Some numerical details are summarized in the last section.

## Model

We consider an orthogonal cubic lattice with 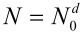 cylindrically symmetric particles positioned at equidistant sites in the space with *d* dimensions. The nearest neighbour’s distance is taken as a unit, thus the side of the cell has the length *L* = *N*_0_. Local orientation of a particle at the site with index *α* is given by a unit vector – director ***S****_α_*. We further set at randomly chosen sites of concentration *p* cylindrically symmetric quenched impurities enforcing orientational ordering along ***e****_α_*. The orientations of impurities are randomly chosen without any preferred global orientation. We also impose a homogeneous external (e.g., electric or magnetic) ordering field ***B*** = *B****e***_B_, which tends to reorient the director field along ***e***_B_. Systems with the head-to-tail invariance, where ±***S****_α_* orientations are equivalent, are taken into account. This property is characteristic for most LC molecules (where several structural details are averaged out via relatively fast molecular rotations) or nanotubes. The corresponding interaction energy of the system can be expressed as [6,8,23]

[1]
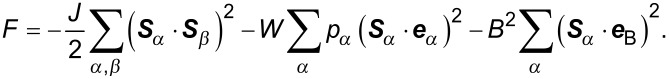


The parameter *J* > 0 describes the ordering interaction among neighbouring molecules tending to orient directors parallel. The index *α* in the double sum counts all the particles, and the indices *β* run over the 1st nearest neighbours of the *α*-th particle. At randomly chosen sites of concentration *p* we additionally place rigid impurities which are coupled with surrounding directors by the random anisotropy type interaction [24–25] of anchoring strength *W* > 0. At the sites with impurities *p_α_* = 1 while at remaining sites *p_α_* = 0.

We describe ordering in the Cartesian coordinate frame (*x*,*y*,*z*), whose axes point along unit vectors **e***_x_*, **e***_y_* and **e***_z_*, respectively. The external field is oriented along a chosen axis, e.g., *x*-axis. We consider behaviour in two and three dimensions, to which we henceforth refer as 2D and 3D, respectively.

For latter convenience we scale quantities in Equation 1 with respect to *J*: 
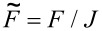
, 
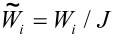
, 
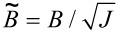
, i.e. we set *J* = 1 in (1). We henceforth omit the tildes. Some details of the minimization of the total energy Equation 1 are given in the numerical approach section. We have neglected the role of thermal fluctuations and consider configurations at zero temperature. In case of nematic ordering in liquid crystals such assumption is sensible deep in the nematic phase (i.e. well below the isotropic-nematic LC phase transition temperature).

In simulations we either originate from randomly distributed orientations of directors, or from homogeneously aligned samples along a symmetry breaking direction. In the latter case the directors are initially homogeneously aligned along **e***_x_*. We henceforth refer to these cases as the i) random and ii) homogeneous samples, respectively. The i) random case can be experimentally realized by quenching the system from the isotropic phase to the ordered phase without an external field (i.e., *B* = 0). This can be achieved either via a sudden decrease of temperature or sudden increase of pressure. The ii) homogeneous case can be realized by applying first a strong homogeneous external field ***B*** along a symmetry breaking direction. After a well enough alignment is achieved the field is switched off.

In order to diminish the influence of statistical variations we carry out several simulations (typically *N*_rep_ ≈ 10) for a given set of parameters (i.e., *W*, *p* and a chosen initial condition).

From obtained configurations we calculate the orientational correlation function *G*(*r*). It measures orientational correlation of LC directors as a function of their mutual separation *r* (*r* = 1 for nearest neighbours). We define it in two dimensional (2D) ensembles as

[2]
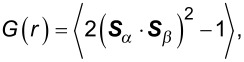


and in three dimensions (3D) as

[3]
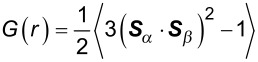


The brackets 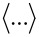 denote the average over all lattice sites that are separated for a distance *r*. If the directors are completely homogeneously aligned along a single direction it follows *G*(*r*) = 1. On the other hand *G*(*r*) = 0 reflects completely uncorrelated directors. Since each director is parallel with itself, it holds *G*(0) = 1. The correlation function is a decreasing function of the distance *r*.

In order to obtain structural details from a calculated *G*(*r*) dependence we use the ansatz

[4]
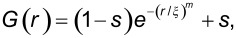


with adjustable parameters *ξ*, *m*, and *s*. The correlation length *ξ* estimates the average domain in which directors are significantly correlated. The parameter *m* measures the distribution width of *ξ* values. Presence of a single correlation length in the system is reflected in *m *≈ 1. A value of *s* reveals the degree of ordering within the system. The case *s* = 0 indicates the short range order (SRO). A finite value of *s* reveals either the long range order (LRO) or quasi long range order (QLRO). To distinguish between these two cases a finite size analysis *s*(*L*) must be carried out where *L* represents the typical linear size of the system. If *s*(*L*) saturates at a finite value the system exhibits LRO. If *s*(*L*) dependence exhibits algebraic dependence on *L* the system possesses QLRO.

## Results

We study the influence of an external ordering field on nematic ordering which is orientationally perturbed by randomly distributed impurities of concentration *p*. We vary the history of samples, concentration *p* of impurities, anchoring strength *W* between LC molecules and impurities, dimensionality of the system and the external field strength *B*. We consider 2D and 3D systems. Concerning histories we either originate from initially homogeneously aligned directors or from completely disordered configuration.

For a given set of control parameters we calculate a configuration of the system by minimizing the interaction energy. The configuration reflects the interplay among elastic, external ordering field and surface disordering tendencies. The external ordering (*B*) and impurities introduce additional characteristic scales into the system. The relative strength of elastic and external ordering field contribution is measured by the external field extrapolation length [7] 
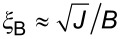
. In the case of ordered LC-substrate interfaces the relative importance of surface anchoring term is measured by the surface extrapolation length [7] *d*_e_ ≈ *J*/*W*. The external ordering field is expected to override the surface anchoring tendency in the limit *d*_e_/*ξ *>>* 1*. However, if LC-substrate interfaces introduce a disorder into the system, then instead of *d*_e_ the so called Imry-Ma scale *ξ*_IM_ characterizes the ordering of the system. It expresses the relative importance of elastic ordering and surface disordering term. It roughly holds [26]

[5]
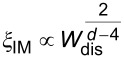


where *W*_dis_ 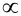 *W* measures the disorder strength.

From obtained orientational ordering we calculate the correlation function *G*(*r*). From it we extract the average correlation length *ξ* using Equation 4. In case that the disorder dominates the system behavior one expects *ξ* ≈ *ξ*_IM_. On the contrary, the dominance of *B* is reflected in *ξ* ≈ *ξ*_B_.

Note that for cases studied we obtain qualitatively similar results for 2D and 3D systems. Consequently, we carry out more detailed simulations for 2D systems which demand less computational time.

**Figure 1:**
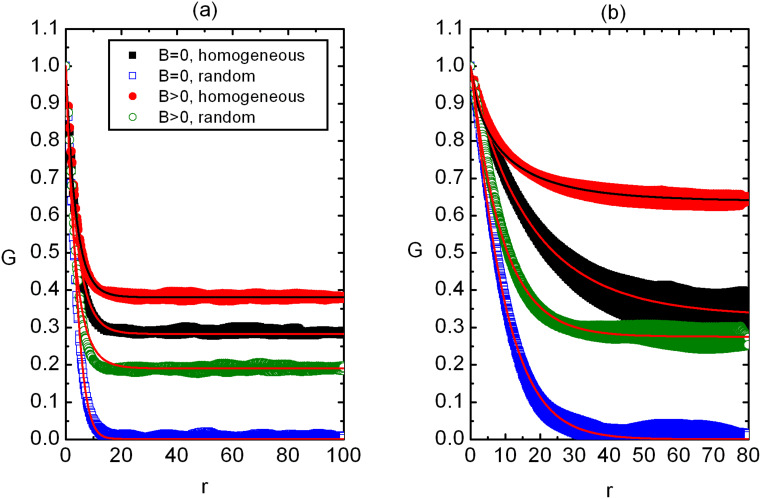
The orientational correlation function as a function of separation *r* between LC molecules for (a) 2D and (b) 3D systems. In random samples *G*(*r*) vanishes for large enough values of *r* for *B* = 0 while in homogeneous samples it could saturate at a finite plateau *s* (if *p* or *W* are low enough). For *B* > 0 a finite plateau can be observed also in random samples. (a) *p* = 0.3, *W* = 2.5, *N*_0_ = 260; (b) *p* = 0.3, *W* = 2.5, *N*_0_ = 100. At *r* = 1 the first neighbors are placed in the cubic cell. The legend is shown in (a).

A typical *G*(*r*) dependence in 2D and 3D is shown in Figure 1a and Figure 1b, respectively. We plot *G*(*r*) for both homogeneous and random initial configuration in the presence of external field and without it. For *B* = 0 it holds *ξ*^(hom)^ > *ξ*^(ran)^, where superscripts (hom) and (ran) denote correlation lengths in homogeneous and random samples, respectively. The reason behind this are stronger elastic frustrations in random samples, as analyzed in more detail in our previous paper [25]. Furthermore, *ξ*^(ran)^ roughly obeys the Imry-Ma scaling for low enough external fields (i.e. *ξ*^(ran)^ << *ξ*_B_), suggesting *ξ*^(ran)^ ≈ *ξ*_IM_. The presence of *B* becomes apparent when *ξ*_≈_ < *ξ*_IM_, which is shown in Figure 1.

We also note that in random samples *s* = *G*(*r*→∞) always equals zero [25] for *B* = 0 indicating short range order. On the contrary in homogeneous samples we obtain a finite value of *s* if the disorder strength is not too large. In Figure 1 we see that the presence of external field can enforce a finite value of *s* also in random samples.

**Figure 2:**
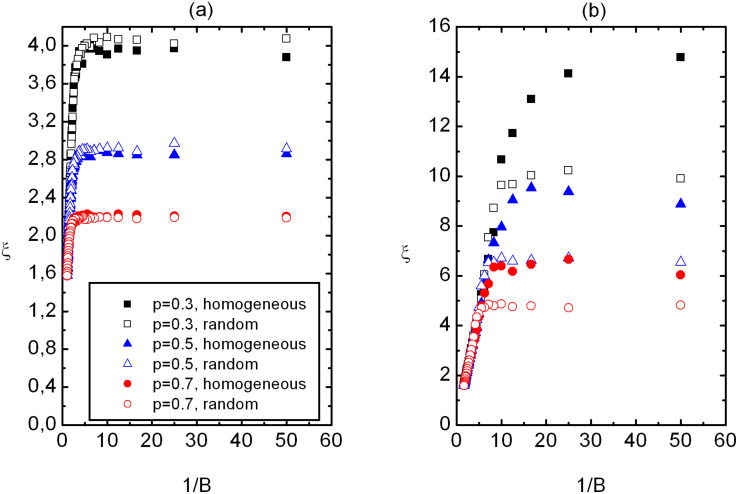
Correlation length *ξ* as a function of 1/*B* for homogeneous and random samples for three different concentrations of impurities in (a) 2D and (b) 3D. The *ξ*_B_(*B*) dependence displays a crossover between the disordered and ordered regime. The disordered regimes extends at (*B* < *B*_c_), where *ξ*^(hom)^ > *ξ*^(ran)^. In the ordered regime (*B* > *B*_c_) one observes *ξ*^(ran)^ ≈ *ξ*^(hom)^ ≈ *ξ*_B_. (a) *W* = 2.5, *N*_0_ = 260; (b) *W* = 2.5, *N*_0_ = 100. The legend is shown in (a).

In Figure 2 we plot *ξ* as a function of 1/*B* for both homogeneous and random samples. For strong enough magnetic fields one expects *ξ* ≈ *ξ*_B_ 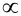 1/*B*. On the other hand for a weak enough *B* the value of *ξ* is dominantly influenced by the disorder strength. Indeed, we observe a crossover behavior in *ξ(B)* dependence on varying *B*. The crossover between two qualitatively different regimes roughly takes place at the crossover field *B*_c_. We define it as the field below which the difference between *ξ*^(ran)^ and *ξ*^(hom)^ is apparent. Below *B*_c_ the disordered regime takes place, where *ξ *exhibits weak dependence on *B*, i.e. *ξ* ≈ *ξ*_IM_. Above *B*_c_ the ordered regime exists, where *ξ* ≈ *ξ*_B_ 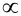 1/*B*. Therefore, for *B* > *B*_c_ it holds *ξ*^(ran)^ ≈ *ξ*^(hom)^ ≈ *ξ*_B_ and in the random regime one observes *ξ*^(hom)^ ≈ *ξ*^(ran)^ ≈ *ξ*_IM_.

**Figure 3:**
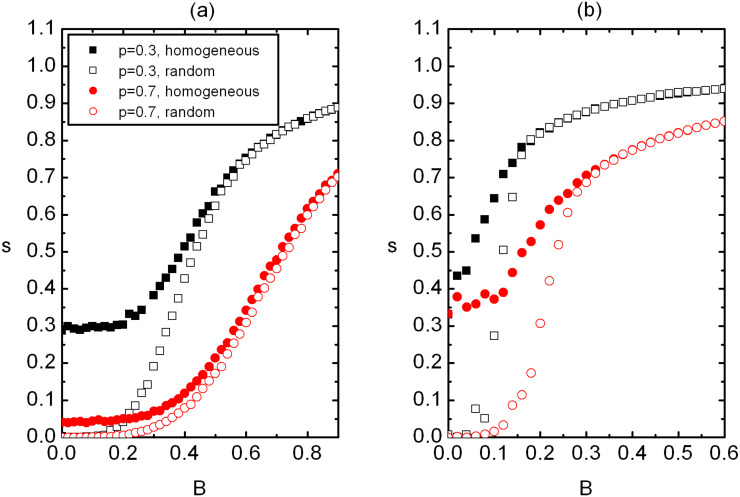
The *s*(*B*) dependence for homogeneous and random samples for two different *p* in (a) 2D and (b) 3D. For *s*(*B* = 0) we obtain *s*^(ran)^ = 0. In the disordered regime it holds *s*^(hom)^ > *s*^(ran)^ and *s*^(hom)^ ≈ *s*^(ran)^ in the ordered regime. (a) *W* = 2.5, *N*_0_ = 260; (b) *W* = 2.5, *N*_0_ = 100. The legend is shown in (a).

The corresponding *s*(*B*) dependence is shown in Figure 3. As expected *s* monotonously increases on increasing *B*, because the external field tends to increase the degree of ordering. Note that in random samples *s*(*B = *0) = 0 and the presence of *B* gives rise to *s* > 0.

**Figure 4:**
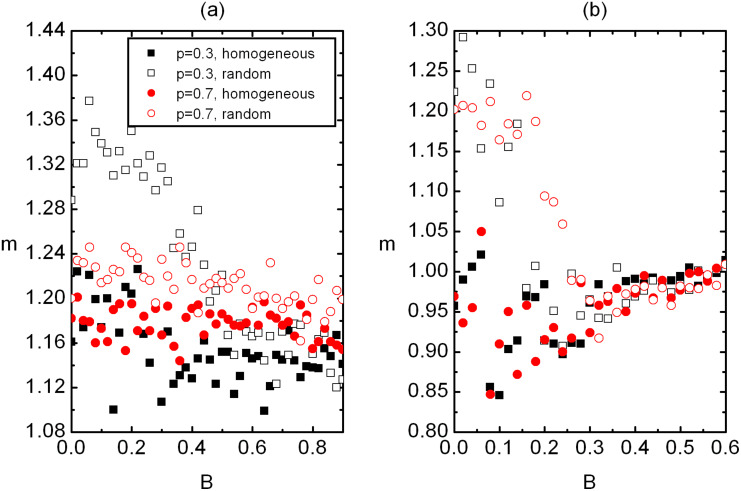
The *m*(*B*) dependence *r* homogeneous and random samples for two different *p* in (a) 2D and (b) 3D. In the disordered regime it holds *m*^(ran)^ > *m*^(hom)^ > 1. In the ordered regime we obtain *m*^(ran)^ ≈ *m*^(hom)^ > 1 which asymptotically approach one on increasing *B*. (a) *W* = 2.5, *N*_0_ = 260; (b) *W* = 2.5, *N*_0_ = 100. The legend is shown in (a).

In Figure 4 we show the *m*(*B*) dependence. For weak enough fields (*B* << *B*_c_) one typically observes *m*^(ran)^ > *m*^(hom)^ > 1. Therefore, in random samples we have larger dispersion of *ξ* values than in homogeneous samples. With the increasing external field both *m*^(ran)^ and *m*^(hom)^ asymptotically approach towards *m* = 1. In the latter case the distribution of *ξ* vales is sharply centered at *ξ* ≈ *ξ*_B_.

**Figure 5:**
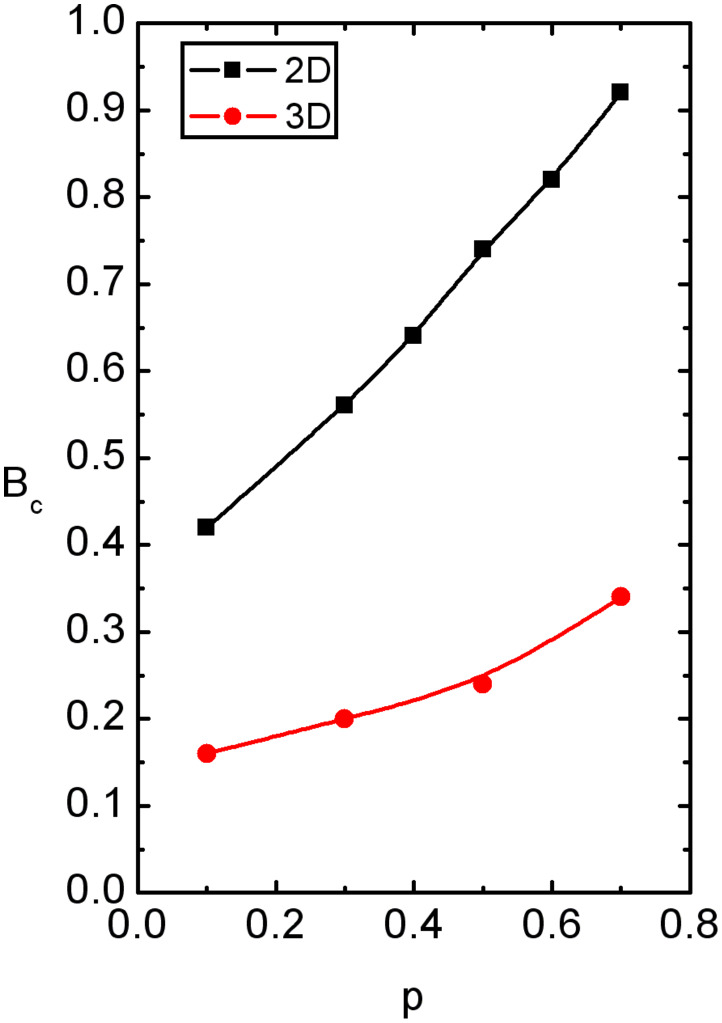
The crossover field *B*_c_ on varying *p*. Indicated lines roughly separate ergodic (*B* > *B*_c_) and nonergodic regimes (*B* < *B*_c_). With increasing *p* one the degree of frustration within the system increases. Consequently larger values of *B* are needed to erase disorder induced memory effects. The points are calculated and the lines serve as guides for the eye. (a) *W* = 2.5, *N*_0_ = 260; (b) *W* = 2.5, *N*_0_ = 100.

The crossover field *B*_c_ as a function of *p* is shown in Figure 5. Indicated lines roughly separate ergodic (*B* > *B*_c_) and nonergodic regimes (*B* < *B*_c_). With increasing *p* the degree of frustration within the system increases. Consequently larger values of *B* are needed to erase disorders induced memory effects. Note that *B*_c_ is larger in 2D than in 3D systems because in the former case the LC molecules are effectively more constrained by impurities (i.e., in 3D the additional degree of freedom is present).

## Conclusions

We have studied the influence of external ordering electric or magnetic field *B* on systems of rod-like objects (e.g. nematic liquid crystals or a dispersion of nano-rods in a liquid environment [20–21]) in the presence of random anisotropic type of disorder. We express the interaction energy *F* of the system using the Lebwohl–Lasher type semi-microscopic description. The orientational order is described in terms of the uniaxial director field exhibiting head-to-tail invariance. We calculate configurations of director fields by minimizing *F* at temperature zero. Therefore, our results are reasonable deep in the nematic phase, where the long range orientational order is observed in absence of random fields. In addition we neglect biaxial states [27–28] which might be present in strongly elastically perturbed systems. For a given set of parameters (i.e. concentration *p* of impurities imposing random anisotropy disorder, disorder anchoring strength *W*, system dimensionality, history of systems and *B*) we calculate the orientational correlation function *G*(*r*) of the system. From it we extract the average size of correlated regions and distribution of *ξ* values measured via the distribution parameter *m*.

Our main interest was to determine the magnetic field regime in which random-field driven memory effects are erased by a strong enough magnetic field. For this purpose we monitored *ξ* dependence on *B* for random and homogeneous initial configurations. These states represent two extreme conditions and consequently yield relatively strong memory effects for weak enough values of *B*. On increasing *B* values of *m* are approaching towards *m* = 1. This signifies that the single peak distribution of *ξ* values is narrowing. On varying *B* we distinguish between two qualitatively different regimes. The *disorded* regime, where random field effects are apparent, exists below *B*_c_. In it we find *ξ*^(hom)^ > *ξ*^(ran)^. In the ordered regime *B* > *B*_c_ the average length *ξ* is dominated by external field *B* and *ξ* ≈ *ξ*_B_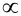 1/*B*. The crossover values *B*_c_ are larger in 2D systems, and monotonously increase on increasing *p*.

The results of our studies suggest regimes in which memory effects are expected. Our settings well mimic for example mixtures of LCs and aerosil particles [13–15] or LCs confined to porous matrices [10–12], or randomly perturbed nanotubes in a liquid environment [20–21]. Our results might be of use for electro-optic applications, where switching between different optical states (i.e. global orientational ordering) is achieved via external electric or magnetic fields in advanced soft nano-composites or soft hybrid systems.

## Numerical approach

The system consists of a lattice of *N*_0_ × *N*_0_ × *N*_0_ sites with unit directors

[6]
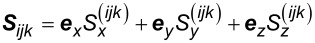


In 2D we set *S*_Z_ = 0. We express the total interaction energy functional as the sum over all sites 
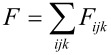
 where the term *F_ijk_* consists of three parts:

[7]
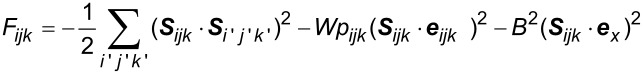


*J* = 1. The indices 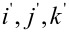 run over the first neighbors of the point described by the indices *I*, *j*, *k*. With respect to denotation of indices in Equation 1 these sets of indices correspond to α = (*I,j,k*) and 
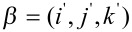
. The parameter *p_ijk_* has the value either 1 or 0, while the orientation of the unit vector ***e****_ijk_* is random spatially distributed, we set these by random-number generator.

The equilibrium director configuration is obtained by minimizing the total interaction energy with respect to all the directors by taking into account the normalization condition 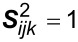. The resulting potential to be minimized reads 
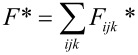
, where

[8]
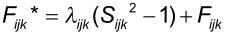


and *λ_ijk_* are Lagrange multipliers. We minimize the potential F^*^ and obtain the following set of 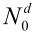
equations which are solved numerically:

[9]
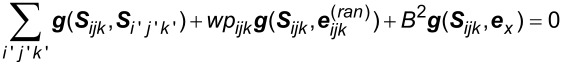


where the vector function ***g*** is defined as

[10]
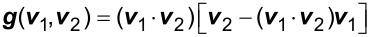


The system of Equation 9 is solved by overrelaxation method which has been proved fast and reliable. At cell boundaries we impose the periodic boundary conditions.
